# Brain Structural Correlates of Emotion Recognition in Psychopaths

**DOI:** 10.1371/journal.pone.0149807

**Published:** 2016-05-13

**Authors:** Vanessa Pera-Guardiola, Oren Contreras-Rodríguez, Iolanda Batalla, David Kosson, José M Menchón, Josep Pifarré, Javier Bosque, Narcís Cardoner, Carles Soriano-Mas

**Affiliations:** 1 Child-Juvenile Mental Health Center of Sant Joan de Déu, Lleida, Spain; 2 Biomedical Research Institute (IRB), Lleida, Spain; 3 Medicine Department, University of Lleida, Lleida, Spain; 4 Department of Psychiatry, Bellvitge University Hospital, Bellvitge Biomedical Research Institute-IDIBELL, and Centro de Investigación Biomédica en Red de Salud Mental (CIBERSAM), Barcelona, Spain; 5 GSS, Hospital Santa Maria, Psychiatry Department, Lleida, Spain; 6 Department of Psychology, Rosalind Franklin University of Medicine and Science, North Chicago, United States of America; 7 Department Clinical Sciences, University of Barcelona, Barcelona, Spain; 8 Medical Department of Ponent Penitentiary Center, Lleida, Spain; 9 Depression and anxiety program, Department of Mental Health, Parc Tauli Sabadell, Hospital Universitari, Barcelona, Spain; 10 Department of Psychiatry and Legal Medicine, Universitat Autònoma de Barcelona, Barcelona, Spain; 11 Department of Psychobiology and Methodology of Health Sciences, Universitat Autònoma, Barcelona, Spain; University of Udine, ITALY

## Abstract

Individuals with psychopathy present deficits in the recognition of facial emotional expressions. However, the nature and extent of these alterations are not fully understood. Furthermore, available data on the functional neural correlates of emotional face recognition deficits in adult psychopaths have provided mixed results. In this context, emotional face morphing tasks may be suitable for clarifying mild and emotion-specific impairments in psychopaths. Likewise, studies exploring corresponding anatomical correlates may be useful for disentangling available neurofunctional evidence based on the alleged neurodevelopmental roots of psychopathic traits. We used Voxel-Based Morphometry and a morphed emotional face expression recognition task to evaluate the relationship between regional gray matter (GM) volumes and facial emotion recognition deficits in male psychopaths. In comparison to male healthy controls, psychopaths showed deficits in the recognition of sad, happy and fear emotional expressions. In subsequent brain imaging analyses psychopaths with better recognition of facial emotional expressions showed higher volume in the prefrontal cortex (orbitofrontal, inferior frontal and dorsomedial prefrontal cortices), somatosensory cortex, anterior insula, cingulate cortex and the posterior lobe of the cerebellum. Amygdala and temporal lobe volumes contributed to better emotional face recognition in controls only. These findings provide evidence suggesting that variability in brain morphometry plays a role in accounting for psychopaths’ impaired ability to recognize emotional face expressions, and may have implications for comprehensively characterizing the empathy and social cognition dysfunctions typically observed in this population of subjects.

## Introduction

Psychopathy is a personality disorder characterized by lack of emotional depth, callous treatment of others, poor judgment and impulsive antisocial behavior [1,2]. Specifically, the emotional behavior of psychopaths is characterized by a reduced sensitivity to punishment and a lack of empathy for other people affected by their behaviors [1,3,4]. Moreover, this condition has been associated with deficits in emotion perception and recognition, featuring impaired recognition of various emotions [2], including deficits in fear and sadness recognition in several studies (for a review, see [2,5,6]). Nevertheless, results have been somewhat heterogeneous, with a recent meta-analysis [2] reporting alterations not being limited to negative emotions, but also extending to the processing of facial and vocal stimuli associated with positive emotions. In addition, two studies reported no alterations in facial emotion recognition in psychopathy [7,8]. The exact pattern of emotional processing alterations observed in psychopathy is therefore not totally understood, and other factors, such as stimulus complexity, have been suggested to play a relevant role [9,10]. Thus, studies using morphed images of facial affect have more consistently found deficits in emotion recognition than studies using static displays [2]. Morphing tasks allow for the assessment of complex emotional stimuli and have been validated for evaluating emotion recognition deficits in samples with psychopathic traits.

Brain imaging tools have been used to obtain information regarding the functional correlates of these emotional face recognition deficits in adult psychopaths. However, the three studies that have investigated such functional correlates have provided mixed findings. These involved both decreased [11,12] and increased [13] activation of frontal and other cortical regions of the face processing brain network, including occipital and fusiform areas, during morphing and static face emotional recognition tasks, respectively. Moreover, Decety and colleagues [12] reported decreased amygdala activation to fear and sadness, together with specific increases in insula activity to fear, sadness and pain facial expressions. In this context, structural imaging data may be particularly valuable to disentangle the mixed findings obtained from functional imaging studies in psychopaths. Morphological alterations in brain regions that have an important role in emotion and social cognition (i.e., the frontal and temporal cortices, the insula and the amygdala) allegedly have a neurodevelopmental root in psychopathic individuals [14–24]. Brain morphological changes might therefore underpin the probably more variable and state-dependent functional alterations in psychopaths during face emotional processing [25,26]. Structural brain correlates of facial emotion recognition in psychopathy, however, have not been previously investigated.

In this study, we used structural Magnetic Resonance Imaging (MRI) and Voxel Based Morphometry (VBM)[27] to evaluate, in comparison with male healthy controls, the relationship between regional gray matter (GM) brain volumes and facial emotion recognition deficits in male offenders with psychopathic traits. To measure emotional processing capacity we selected a morphing facial emotion recognition task [28,29]. We anticipated that psychopathic individuals would show deficits in recognizing both negative and positive emotional face expressions, although with larger difficulties in recognizing sad and fearful expressions. At the imaging level, based on findings from previous functional imaging studies assessing emotional face recognition deficits in psychopaths [11–13] and the arguably use of frontal and visual cortices as compensatory resources during emotional processing [13,30–33], we predicted associations between recognition of facial emotional expressions and gray matter volumes of the dorsomedial and lateral prefrontal, as well as occipital, cortices. Associations with structural changes in emotion processing regions such as the amygdala and the insula may also be observed, although this hypothesis is based on a limited amount of evidence from functional imaging literature [12].

## Material and Methods

### Participants

A total of 19 men with psychopathy (according to Hare criteria [1]), with a documented history of severe criminal offense, were assessed and compared with a non-offender control group of 20 men. Sample characteristics are fully described in Table 1, S1 Appendix and previous reports [13,24,34].

**Table 1:** Characteristics of study groups.

	Psychopaths	Controls
Age (years), mean ± SD (range)	39.2 ± 8.9 (28–64)	40.6 ± 9.9 (28–61)
Gender	19 men	20 men
Vocabulary WAIS-III (range)	12.1 ± 2.8 (7–18)	10.2 ± 2.3 (6–14)
Education (years), mean ± SD (range)	9.2 ± 2.8 (4–14)	10.6 ± 2.3 (8–16)
Handedness (left/right)	1/19	1/20
PCL-R total, mean ± SD (range)	*28.1±3.6 (22.2–34.4)	0.5 ± 0.9 (0–3)
PCL-R factor-1, mean ± SD (range)	*12.7 ± 2.3 (8–16)	0.2 ± 0.5 (0–2)
PCL-R factor-2, mean ± SD (range)	*13.4 ± 4.3 (5–20)	0.3 ± 0.6 (0–2)
Comorbidities:		
DSM-IV-R Axis I diagnosis ^a^	None	None
HRSD score, mean ± SD (range)	*1.9 ± 2.2 (0–8)	0.4 ± 1.0 (0–4)
HAM-A score, mean ± SD (range)	1.9 ± 3.2 (0–10)	0.7 ± 0.8 (0–2)
Y-BOCS total score, mean ± SD (range)	0.6 ± 2.4 (0–10)	0 ± 0 (0–0)
Current substance abuse	None	None
DSM-IV-R Axis II diagnosis ^b^	None	None
Barrat Impulsiveness Scale, total score	*53.2±23.4 (16–103)	32.2±14.5 (16–72)
Torrubia’s Sensivity to Punishment ^c^	8.2 ± 5.7 (0–19)	5.3 ± 4.5 (0–15)
Torrubia’s Sensivity to Reward ^c^	11.9 ± 5.5 (5–22)	6.7 ± 4.6 (0–20)

* indicates p< 0.01.

^a^ Except past history of substance abuse.

^b^ Except Antisocial Personality Disorder.

^c^ Sensitivity to Punishment and Sensitivity to Reward Questionnaire.

WAIS-III, Wechsler Adult Intelligence Scale (third edition); HRSD: Hamilton Depression Rating Scale; HAM-A: Hamilton Anxiety Rating Scale; PCL-R: Psychopathy Checklist-Revised; Y-BOCS: Yale-Brown Compulsive Scale.

Subjects with psychopathy were derived from a total sample of 105 convicted subjects that were initially evaluated using a comprehensive clinical protocol. This sample showed a mean Psychopathy Checklist-Revised (PCL-R) score [1] of 27.8, and served to select individuals for the present study according to the following criteria: (i) a total PCL-R score greater than 25 or a PCL-R Factor 1 score greater than 10, (ii) documented severe criminal offense, (iii) absence of DSM-IV Axis I diagnosis [35] with the exception of past history of substance abuse, (iv) absence of DSM-IV Axis II diagnosis, apart from antisocial personality disorder, (v) absence of symptomatic medical and neurological illness, (vi) normal IQ according to the Wechsler Adult Intelligence Scale-Third Edition-Revised (WAIS-III-R [36]), and (vii) obtainment of subject-specific full administrative permission and special police custody during the day of MRI assessment. Additional information regarding offense history, substance use and abuse, and medical records are reported in Supplementary Material.

The sample of healthy non-offender subjects was recruited from the community and hospital staff. Controls participants were selected to match participants in the psychopathy group in age, sex and Vocabulary scores, and also underwent a comprehensive medical and psychiatric assessment (see Table 1).

The investigation was carried out in accordance with the Declaration of Helsinki. All cases and control subjects gave written informed consent after receiving a complete description of the study, which was approved by two local research and ethics committees (IMIM Hospital del Mar, Barcelona, and Hospital Universitari Arnau de Vilanova, Lleida). Moreover, recruitment and all procedures involving subjects with psychopathy were also approved and monitored by the General Secretariat of Penitentiary Institutions of Catalonia's government. Importantly, it is widely recognized that subjects with psychopathy have preserved cognitive and volitional capacities [1], and therefore are able to provide informed consent. However, the presence of psychopathy does not exclude other conditions (psychiatric o neurologic) that can diminish capacity, which were ruled out as mentioned above for the inclusion and exclusion criteria. Nevertheless, because of the loss of freedom inherent to incarceration, informed consent included a specific excerpt to guarantee voluntary participation.

### Psychopathy assessment

Information for rating the PCL-R was collected by a trained senior psychiatrist from a comprehensive semi-structured interview with the inmate and a review of his institutional files and all available additional information. For the PCL-R, each of the 20 items was scored 0, 1, or 2, depending on the degree to which each disposition was exhibited. The Spanish version of the PCL-R was used [37]. The internal consistency of the assessment was tested for the whole 105-subject sample obtaining a Cronbach’s alpha coefficient of 0.79 (inter-item correlation mean, 0.36) for PCL-R total score.

### Facial emotion expression recognition task

Our task was a slightly modified version of a facial emotion expression recognition task widely used in different research studies [28,29]. Specifically, our task consisted of photographic quality pictures of 12 faces (six men and six women) expressing the six different basic emotional facial expressions of happiness, surprise, fear, sadness, disgust and anger, with all pictures being selected from the NimStim Set of Facial Expressions [38]. Each trial started with a neutral face that gradually morphed through ten stages, in 10% increments, into one of the six prototypical emotional expressions. Participants were asked to watch the emotional expression displayed on the screen and indentify the emotion as soon as they recognize it without merely guessing.

Task performance was scored according to the number of steps required before successful recognition (e.g. successful recognition with 30% of emotion expression would score 3 points). Accordingly, better performance in emotion recognition involved scoring fewer points. A failure to recognize the expression scored 11 points. The experimental session consisted of 12 trials (two per emotion), and a mean expression recognition score for each of the six emotions was obtained by averaging the scores for the two trials of each emotion.

### MRI Acquisition and Preprocessing

A 1.5-Tesla Signa Excite system (General Electric, Milwaukee, Wisconsin, USA) equipped with an eight-channel phased-array head coil and single-shot echo planar imaging software was used. The anatomic sequence consisted of high-resolution axial T1-weighted anatomic images acquired for each subject using a three-dimensional fast spoiled gradient inversion recovery prepared sequence. Acquisition parameters were 134 contiguous slices (repetition time = 11.8 msec, echo time = 4.2 msec, flip angle = 15°, field of view = 30 cm, 256 x 256 pixel matrix, slice thickness = 1.2 mm).

Preprocessing and statistical analysis of the anatomic imaging data were conducted using MATLAB version R2008b (The MathWorks, Inc, Natick, Massachusetts) and statistical parametric mapping software (SPM8; Wellcome Trust Centre for Neuroimaging, UCL, London, United Kingdom). We used the VBM8 Toolbox for image preprocessing (http://dbm.neuro.uni-jena.de/vbm.html), which involved: 1) bias correction, 2) optimal tissue classification using nonlinear deformation fields to obtain tissue probability maps of gray matter (GM) based on the ICBM Tissue Probabilistic Atlas (http://www.bmap.ucla.edu/portfolio/atlases/ICBM_Probabilistic_Atlases/) that best overlaid the images of the individual subjects (rather than assuming stationary prior probabilities), and 3) image registration using linear (12-parameter affine) and nonlinear transformations (warping) [39]. To restore volumetric information [40,41], the normalized GM images were modulated with Jacobian determinants (derived from the spatial normalization step). Finally, the volumetric images were smoothed with an 8mm full width at half maximum Gaussian kernel. Before tissue classification we checked each individual image for acquisition artifacts and gross anatomical abnormalities. Moreover, accuracy of spatial warping was confirmed by assessing the level of overlap among the different tissue classes for each of the study subjects.

### Statistical Analyses

#### Behavioral performance

Task performance was assessed with an analysis of covariance (ANCOVA) with group as the between-subject factor (psychopaths, controls) and emotion category as the within-subject variable (happy, surprised, disgusted, angry, sad, fearful). Likewise, an independent ANCOVA was performed to assess potential differences in the number of errors to identify each emotion. These analyses were adjusted for potential confounding factors; namely: age, IQ, drug and alcohol abuse, viral infection (HIV, hepatitis B and C) and psychotropic medication, which were used as covariates. Within each emotion category, between-group differences were assessed post-hoc with independent sample t-tests. Cohen effect size was also calculated for each emotion category [42]. Likewise, pair-wise comparisons between each emotion category were performed with paired sample t-tests. Statistical Package for the Social Sciences (SPSS) version 19.0 software (IBM, Chicago; 2010) was used in all the analyses.

#### Structural brain correlates

To investigate the differential anatomical correlates subserving emotional face recognition deficits in psychopaths relative to controls, whole-brain voxel-wise correlation analyses were performed using a two-sample t-test model in SPM8. Specifically, we performed a whole-brain between-group comparison of the voxel-wise correlation scores between the number of steps required to correctly identify emotions and regional GM volumes. We first conducted these analyses only for those emotion categories in which psychopaths showed significant emotional recognition deficits relative to controls. Then, so as to investigate whether differences in brain structure were associated with overall performance in facial affect recognition, we conducted a second analysis including all six emotion categories, regardless of whether groups differed in task performance. The six covariates previously used in behavioral analyses, together with individuals’ global gray matter volume, were included in all imaging analyses. Moreover, we also extracted 3-mm-radius spheres from the peak coordinates of significant between-group differences in correlation analyses to compare regional gray matter volumes within these regions between the study groups. Likewise, groups were compared in global gray matter and total intracranial brain volume. These last analyses were performed in SPSS.

#### Association with PCL-R psychopathy severity scores

Additional correlation analyses were conducted in SPSS to assess for associations between PCL-R facet scores and both emotion recognition scores and regional gray matter volumes. For this last analysis, we extracted the eigenvalues from the peak coordinates of significant clusters from the above between-group comparisons of correlation strengths.

#### Thresholding Criteria

Behavioral analyses were explored at a standard threshold of *p*<0.05. Significance in imaging analyses was established by means of a combination of voxel-level and spatial extent thresholds. Voxel threshold significance was set at *p*<0.01, while spatial extent threshold was determined by 1000 Monte Carlo simulations using the AlphaSim algorithm as implemented by the SPM REST (Resting-State fMRI Data Analysis Toolkit) toolbox [43]. The voxel-level alpha value was set at p<0.01 taking into account the sample size of the study and to avoid compromising the sensitivity of the statistical analyses [44]. In addition, input parameters to AlphaSim included a cluster connection radius of 5 mm and the actual smoothness of the data, incorporating a gray matter mask volume of 167.265 voxels (2 mm x 2 mm x 2 mm). The minimum cluster size was determined to be 1592 mm^3^ (corresponding to 199 voxels) to satisfy a family-wise error (FWE) rate correction of *p*_FWE_<0.05. However, the resulting cluster extent was further adjusted to account for the non-isotropic smoothness of structural images, in accordance with Hayasaka and colleagues [45].

In the correlation analyses between PCL-R facet scores and emotion recognition scores we applied a Bonferroni correction for multiple testing with significance therefore being set at [*p* = 0.05/(6 facial emotions x 4 PCL Facets) = 0.002]. Bonferroni correction was also used for assessing significance of the correlations between PCL-R scores and regional gray matter volumes, and, thus, in these analyses, significance was set at [*p* = 0.05/(15 brain regions (see below) x 4 PCL Facets) = 0.0008].

## Results

### Behavioral performance

The ANCOVA analysis showed a main effect of group (*F*_(1,31)_ = 11.91, *p* = 0.002), indicating that psychopaths required more steps to correctly identify emotions. Main effect of emotion type showed a trend to significance (*F*_(5,155)_ = 2.08, *p* = 0.07), although group x emotion type interaction was not significant (*F*_(5,155)_ = 0.30, *p* = 0.91). We did not observe any significant results when assessing the variable number of errors committed during facial emotion recognition.

Despite the lack of a significant interaction between variables, because of our a-priori hypothesis of a specific impairment for some particular emotions, we performed post-hoc comparisons between psychopaths and healthy controls in each emotion category. Groups differed in recognition accuracy of sad [*t* = 2.393, *p* = 0.023], happy [*t* = 2.423; *p* = 0.021] and fearful [*t* = 3.356; *p* = 0.002] expressions. In all cases psychopathic offenders needed more steps than controls to correctly identify facial emotions. As shown in Table 2, effect sizes were large for happiness, sadness and fear, but also for disgust (all *d*s > 0.70), although in this last emotion we did not observe a statistically significant effect. Given the near significant effect of emotion type, and for the sake of completeness, we also assessed pair-wise differences between each emotion category by means of paired sample t-tests. These data are presented in S1 Table in the supplementary material.

**Table 2:** Emotional face task performance. Mean number of steps needed to recognize each particular emotion ±SD.

	Psychopaths	Controls	*t*	*p*	*d*
Sadness	5.18 ± 1.64	3.90 ± 0.84	5.7	0.023*	0.98
Fear	5.61 ± 1.80	4.47 ± 1.02	11.3	0.002*	0.77
Happiness	3.13 ± 0.98	2.28 ± 0.85	5.9	0.021*	0.92
Surprise	4.92 ± 1.91	4.18 ± 1.51	2.9	0.096	0.43
Anger	4.03 ± 1.89	3.08 ± 1.20	2.5	0.128	0.60
Disgust	10.34 ± 2.43	8.20 ± 2.94	2.6	0.118	0.79

*p<0.05.

*d* = Cohen effect size

### Structural brain correlates

#### Sadness recognition

**Fig 1:**
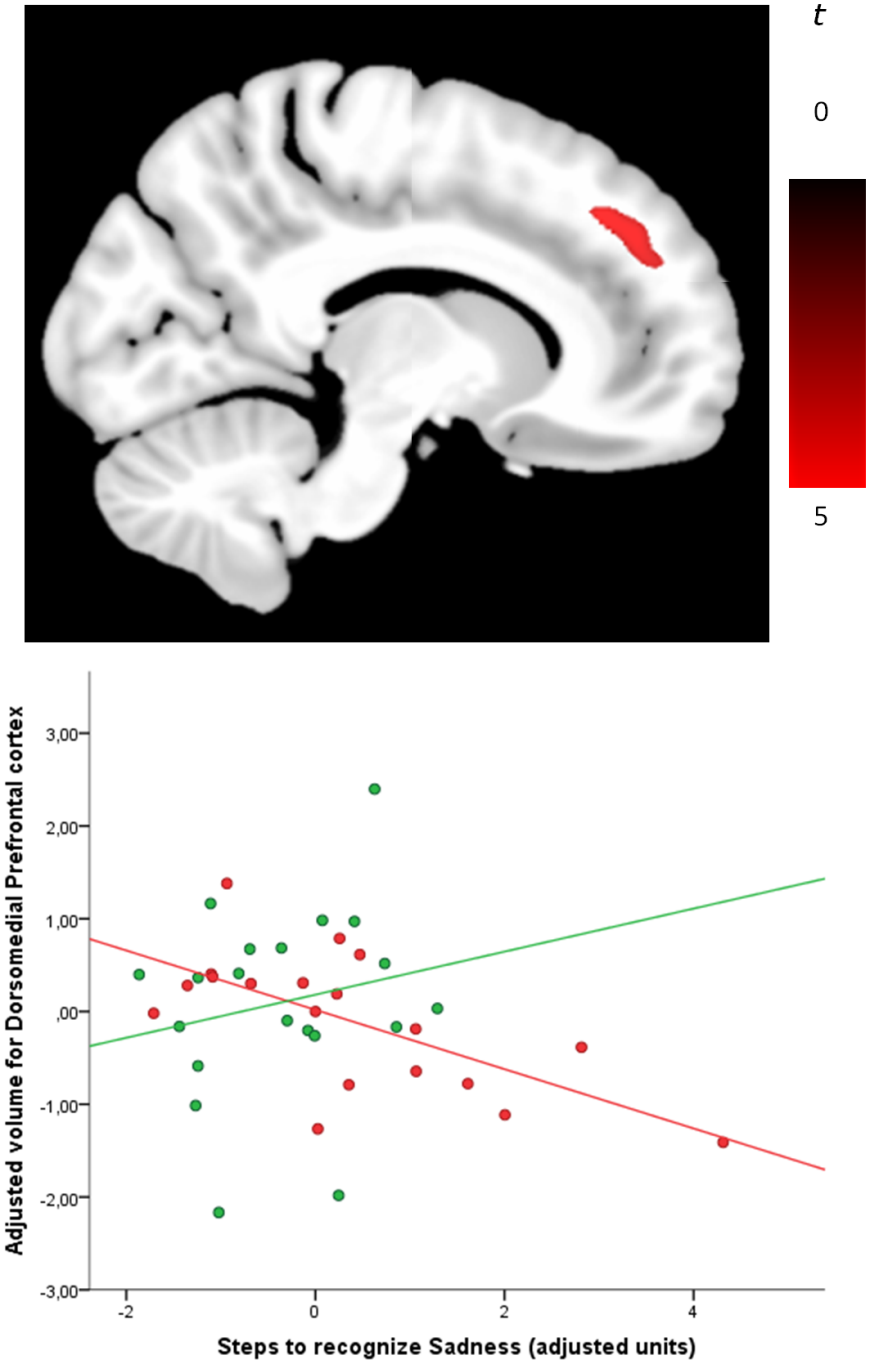
The specific contribution of regional GM volumes in the dorsomedial prefrontal cortex (MNI coordinates, x = 17, y = 43, z = 33) to improved sadness recognition in psychopaths was explained by a negative association in this group (red) but a positive association in controls subjects (green). The plot displays residual values once covariates were controlled for. The sagittal display corresponds to the right hemisphere.

Significant between-group differences in the correlations between regional GM volumes and sadness recognition score were observed in the dorsomedial prefrontal cortex. Specifically, in psychopaths relative to controls, better sadness recognition was associated with greater GM volume in this brain region (see Fig 1 and Table 3).

**Table 3:** Brain volumetric correlates of between-group differences in facial emotion recognition of sad, happy and fear expressions.

Brain Region		x, y, z	t	CS
Sad				
Controls > Psychopaths				
Dorsomedial PFC	*right*	17, 43, 33	2.7	240
Happy				
Controls > Psychopaths				
Middle ACC	*left*	-6, 2, 41	3.6	241
Inferior Frontal gryus	*right*	59, 23, 15	4.3	364
Orbitofrontal cortex	*right*	47, 41, -4	3.2	241
Insula (anterior)	*left*	-39, 15, -9	3.6	285
Cerebellum	*right*	17, -36, -24	4.6	1743
Psychopaths > Controls				
Precentral gyrus	*right*	56, 6, 23	3.9	234
Insula (posterior)*	*left*	-48, -18, 9	3.8	580
Amygdala	*right*	29, -2, -17	3.6	237
	*left*	-14, -3, -16	3.3	543
Fear				
Controls > Psychopaths				
Somatosensory cortex	*left*	-26, -29, 63	3.5	359
Psychopaths > Controls				
Middle ACC	*left*	-3, 3, 41	3.5	352
Temporal cortex	*right*	50, -48, 9	4.5	529
	*left*	-48, -42, 12	3.8	276

Coordinates (x, y, z), in Montreal Neurological Institute (MNI) Atlas space, correspond to the local maxima inside each of the brain clusters that survived our thresholding criteria. Abbreviations: PFC: Prefrontal cortex, ACC: Anterior cingulate cortex. CS: Cluster size.

*Brain region showing decreased volume in psychopaths.

#### Happiness recognition

**Fig 2:**
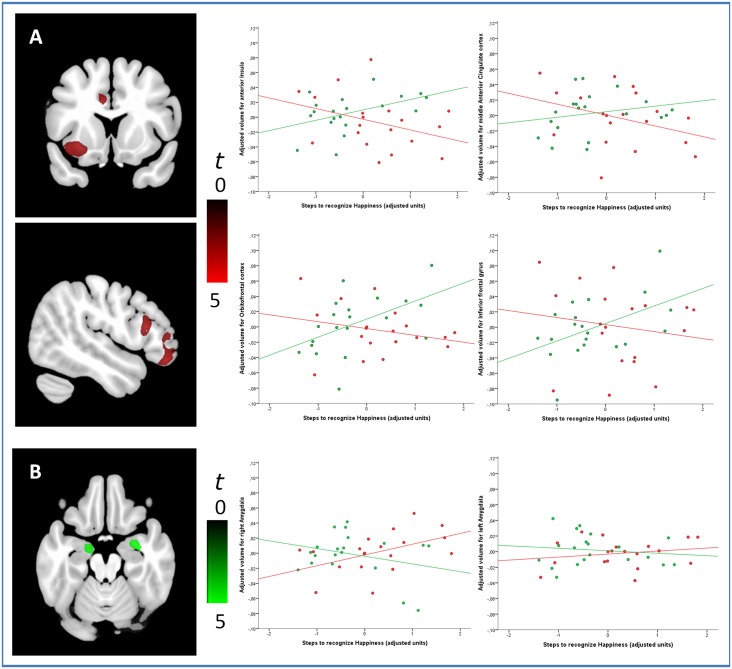
(A) The specific contribution of regional GM volumes in the anterior insula, middle anterior cingulate cortex, orbitofrontal cortex and inferior frontal cortex (Table 3) to improved happiness recognition in psychopaths was explained by a negative association in this group (red) but a positive association in controls subjects (green). (B) Conversely, the specific contribution of regional GM volumes in the amygdalae (Table 3) to improved happiness recognition in control subjects was explained by a negative association in this group (green) but a positive association in psychopaths (red). The plot displays residual values once covariates were controlled for. Images are displayed in neurological convention, therefore the right hemisphere corresponds to the right side in axial and coronal displays. The sagittal display corresponds to the right hemisphere.

Significant between-group differences in the correlations between regional GM volume and happiness recognition score were observed in the middle anterior cingulate cortex, anterior and posterior insula, the inferior frontal gyrus, the orbitofrontal cortex, bilateral amygdala, the precentral gyrus and the anterior lobe of the cerebellum. Specifically, in psychopaths relative to controls, better happiness recognition was associated with greater GM volume in the middle anterior cingulate cortex, anterior insula, inferior frontal gyrus, orbitofrontal cortex and anterior cerebellum. Controls, in turn, showed better task performance associated with greater GM volume in the precentral gyrus, posterior insula and in bilateral amygdala (see Fig 2 and Table 3).

#### Fear recognition

**Fig 3:**
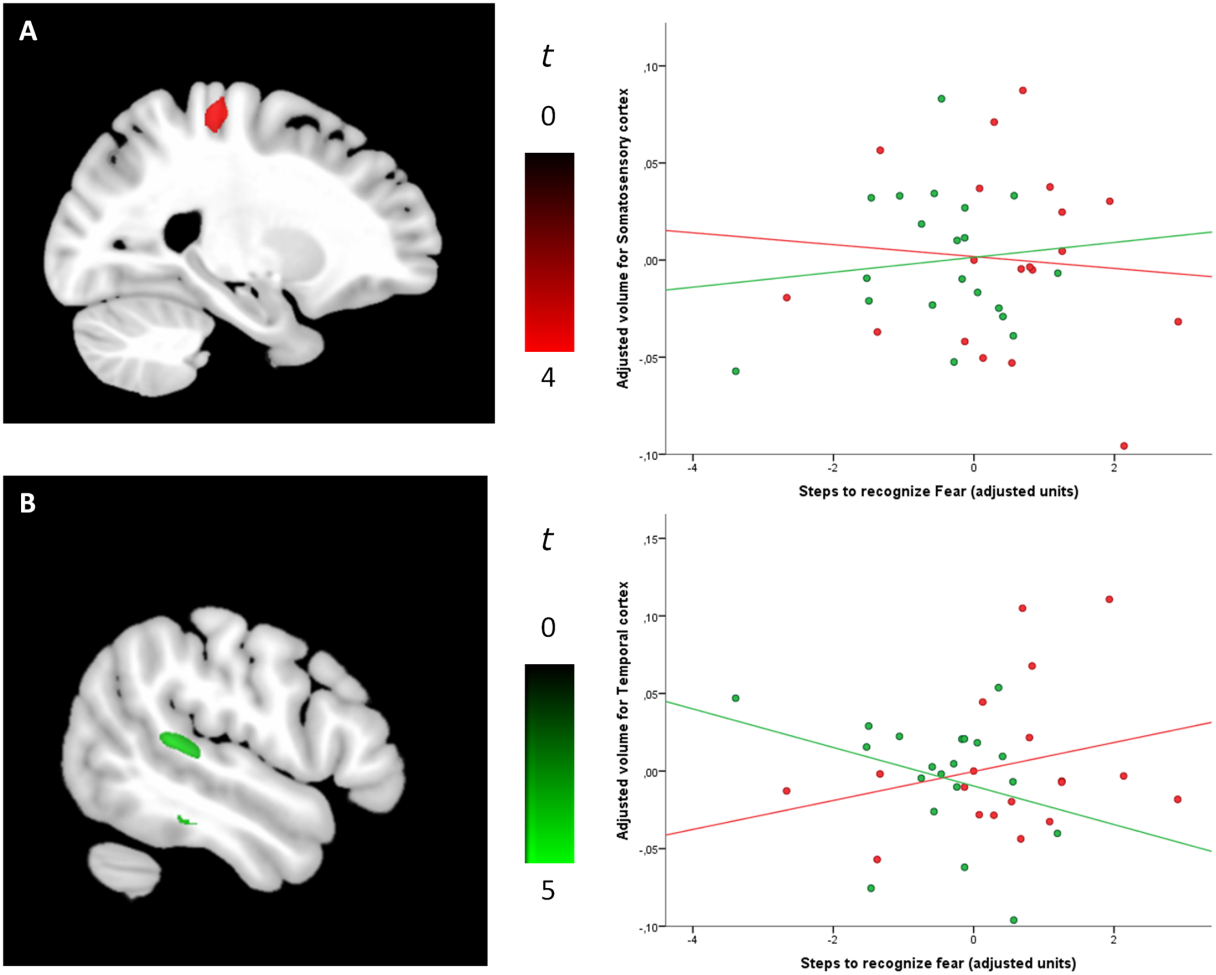
(A) The specific contribution of regional GM volumes in the somatosensory cortex (MNI coordinates, x = -26, y = -29, z = 63) to improved fear recognition in psychopaths was explained by a negative association in this group (red) but a positive association in the control subjects (green). (B) Conversely, the specific contribution of regional GM volumes in the temporal cortex (MNI coordinates, x = -48, y = -42, z = 12) to improved fear recognition in controls subjects was explained by a negative association in this group (green) but a positive association in psychopaths (red). Plots display residual values once covariates were controlled for. The sagittal displays correspond to the left hemisphere.

Significant between-group differences in the correlations between regional GM volume and fear recognition scores were observed in the somatosensory cortex, middle anterior cingulate cortex and the superior temporal gyrus. Specifically, in psychopaths, relative to controls, better fear recognition was associated with greater GM volume in the somatosensory cortex. Controls, in turn, showed better task performance associated with greater GM volume in the middle anterior cingulate cortex and superior temporal gyrus (see Fig 3 and Table 3).

#### Overall emotional face recognition

Collapsing across all emotion categories, significant between-group differences were observed in the correlation between emotion recognition score and regional GM volume in the amygdala, posterior insula, parahippocampus and the posterior lobe of the cerebellum. Specifically, in psychopaths, relative to controls, better fear recognition was associated with greater GM volume in the cerebellum. Conversely, in controls, better task performance was associated with greater GM volume in the amygdala, posterior insula, and parahippocampus (see S1 Fig and S2 Table).

#### Between-group differences in regional GM volumes

Between group comparisons of the regional GM volumes from the above regions showed that psychopaths, relative to controls, only displayed decreased GM volumes in the posterior insula (Table 3). There were no significant between-group differences for the remaining clusters. Groups did not differ in total gray matter and total intracranial brain volumes either.

### Associations with PCL-R facet scores

In psychopaths, better recognition of sadness was associated with scores on PCL-R facet 3 (*r* = -0.65, *p* = 0.017), and better recognition of fear was similarly associated with scores on PCL-R Facet 4 (*r* = -0.63, *p* = 0.021) (see S2 Fig). However, such associations did not survive the Bonferroni correction for multiple testing. At the imaging level, dorsomedial prefrontal cortex volume (see Table 3) was positively associated with the PCL-R Facet 3 –Lifestyle score (*r* = 0.51, *p* = 0.026; S2 Fig), although, again, this association did not survive the Bonferroni correction.

## Discussion

Psychopath offenders showed deficits in the recognition of sad, happy and fear emotional expressions. In agreement with our hypotheses, subsequent brain imaging analyses showed that psychopaths with better recognition of facial emotional expressions had higher volume in the prefrontal (orbitofrontal, inferior frontal gyrus, dorsomedial prefrontal cortex) and other cortical areas (somatosensory cortex). Findings also showed that GM volumes in the amygdala and temporal cortices contributed to the correct recognition of emotional face expressions in controls only.

Our findings of impaired recognition of emotional face expressions of fear and sadness are in general agreement with findings from previous studies [46–49]. Moreover, in agreement with the results reported here, Hastings and colleagues [50] found deficits in the recognition of happiness facial expressions using a similar morphed recognition task. In the context of emotion-based theoretical accounts of psychopathy, our finding of impaired recognition of fear may be partly in line with the low-fear hypothesis [51–53]. Similarly, the finding of impairment in both fear and sadness seems to be consistent with the Integrated Emotion System (IES) model [54]. However, the preserved recognition of other threatening emotions (i.e., anger [55]) and the deficits in recognizing non-threatening emotions (i.e. sadness and happiness) more likely suggest a blunted overall capacity for experiencing emotions, as has been suggested by some authors [56], with these not necessarily being restricted to the processing of negative emotions (Violence Inhibition Mechanism, VIM [57,58]). Such overall emotion recognition deficits are also supported by the medium to large effect sizes found for all emotions, except for surprise. Notably, the large effect size found for disgust is in agreement with a previous study that demonstrates a specific deficit in classifying disgust in psychopathic individuals [59], although we did not obtain a statistically significant deficit in the recognition of disgust. Nevertheless, deficits in disgust recognition may have been undermined by the fact that this emotion was the most difficult to recognize across groups.

The contribution of greater prefrontal and cortical volumes to better emotional recognition is in agreement with the hyperactivation of these brain areas during an emotional face-matching task in a previous study with this same sample of psychopaths [13]. However, through the use of more complex morphing tasks, other studies found a reduced rather than enhanced activation in prefrontal cortex and the facial face processing brain network, including the fusiform and extrastriate cortices, of psychopaths [11,60]. Discrepancies across such findings may therefore be understood by taking task difficulty into account. Increased volume of prefrontal and other cortical regions may help psychopaths to perform simple emotional tasks, although such compensatory mechanisms may become insufficient in complex tasks. This interpretation resonates with the Response Modulation Hypothesis (RMH; [61]) which suggests that emotion-processing deficits in psychopaths more likely manifest in complex contexts, as opposed to simple contexts where emotional processing can be preserved.

The volume of other brain areas additionally contributed to better emotional face recognition in psychopaths. Firstly, the contribution of anterior insula volumes to better happiness recognition in psychopaths is consistent with two prior functional studies that showed greater activation of the insula during emotional face recognition in psychopaths [11,60]. This finding may reinforce putative contributions of the emotional/empathic subregion of the insula to better emotional face recognition. Secondly, the volumes of the middle cingulate cortex did also contribute to emotional face recognition accuracy in psychopaths. Decety and colleagues [60] reported enhanced activation of the middle portion of the cingulate gyrus in high relative to medium/low psychopaths, and both the insula and the cingulate cortex are part of the paralimbic system, which has shown functional and anatomical alterations in psychopaths [31]. These findings may therefore suggest that the morphological integrity of this system aid in emotional face recognition in psychopaths. Finally, consistent with the activation of the cerebellum when facing diverse emotional face expressions [62], psychopaths with greater volume in this region showed better overall face emotional recognition and, in particular, better recognition of happy expressions. Importantly, this association was observed within the posterior lobe of the cerebellum, which is more likely to be involved in both cognitive and emotional processing, but not within the anterior sections linked to sensorimotor functions [63].

The specific association between amygdala and temporal cortex volumes and overall and happy face recognition in controls relative to psychopaths support the prevailing hypothesis that psychopaths display a dysfunctional emotional-limbic system [54]. Indeed, abnormal amygdala response has been found in adult psychopaths [60] and in samples with psychopathic traits during emotional face recognition tasks [5,64–66]. The lack of association between amygdala and temporal cortex structure and facial emotion recognition observed here in psychopaths may be a specific feature of this patient group, since individuals with mental disorders (e.g., schizophrenia and autism) typically present changes in the amygdala and temporal regions in association with facial emotional processing deficits [67–70]. The fact that amygdala volume was significantly associated with better happiness recognition in controls, but not with fear recognition, may seem somewhat unexpected based on the evidence of amygdala involvement in human fear responding [62,71,72]. However, not all studies have reported a preferential association between the amygdala and the processing of fear [73–76] and neuroimaging studies have shown that the amygdala respond to a range of emotional expressions [77]. Likewise, Zhao and colleagues [78] showed that left amygdala volumes were negatively associated with the accuracy to recognize fearful facial expressions in healthy controls. Also, patients with bilateral amygdala damage present with pervasive emotion recognition deficits [79].

This study has several strengths. Firstly, we have assessed, to our knowledge for the first time, the brain anatomical correlates underpinning emotional face recognition deficits in adult psychopaths, which may help in the interpretation of the mixed findings derived from previous functional neuroimaging research. Moreover, psychopathic individuals were carefully assessed to ensure they displayed core emotional dysfunction and the potential effects of drug use and other confounders were ruled out. By contrast, a limitation of this study is that subjects with psychopathy, unlike controls, were convicted prisoners and so potential effects of incarceration on brain anatomy were not controlled for. However, our findings are not likely to be attributable to between-group differences in absolute or regional gray matter volumes since only the posterior insula showed a significant volume decrease in psychopaths and groups were comparable in global gray matter and total intracranial volume. Furthermore, an inherent limitation of VBM analyses is that this approach, despite providing information about cortical and subcortical structures with a similar level of spatial resolution, is not able to provide information about the precise nature of the cortical alterations (i.e., changes in volume vs. changes in thickness), which could help to further assess the contribution of prefrontal structural integrity to emotional face recognition in psychopaths. In addition, another limitation of this study is that, although groups had comparable errors during emotional face recognition, the absence of response latency data in the morphing recognition task precludes examination of whether the poor accuracy demonstrated by psychopath offenders is secondary to faster and more impulsive responses to affective stimuli [59,79]. Likewise, our study cannot ascertain the real contribution of brain morphological changes in explaining functional alterations during emotional face processing in psychopaths. In this sense, future studies combining functional and anatomical brain measurements should be encouraged to better describe the precise nature of functional alterations and the possible causes of variability across studies. Finally, future studies with larger samples may better investigate the role of antisocial features in improving emotional face recognition in psychopaths, and the corresponding anatomical substrates. In the present study, results regarding this issue were only marginally significant.

In summary, our results provide preliminary evidence of the significant role of brain morphology measurements to fully understand face emotion recognition deficits in psychopaths. These data should also contribute to better appreciate how morphological alterations in psychopaths may relate with the abnormalities in empathy and social behavior domains typically described in this population of subjects [14,16].

## Supporting Information

S1 Appendix Additional characteristics of the psychopath group. (DOCX)

S1 Fig Display of the gray matter brain volumes associated with emotional recognition. (DOCX)

S2 Fig Associations of PCL-R Facets and gray mater volume, and emotional recognition performance. (DOCX)

S1 Table Pair-wise differences between each emotion category. (DOCX)

S2 Table Brain coordinates of the gray matter brain volumes associated with emotional recognition. (DOCX)
