# Supplementary material for: Brain Structural Correlates of Emotion Recognition in Psychopaths
Source: PLoS One. 2016 May 13;11(5):e0149807. doi: 10.1371/journal.pone.0149807 (PMC4866737; doi:10.1371/journal.pone.0149807)
Supplement: S1 Appendix — (DOCX) [file pone.0149807.s001.docx]

**S1 Appendix. Additional characteristics of the psychopath group**

**Offense history**

All 19 individuals were incarcerated in correctional institutions situated in Catalonia (Spain). Mean (± SD) completed incarceration time at inclusion was 85 ± 59 months, range 12-251 months. Mean time of accumulated sentences was 243 ± 131 months; ranging 96-546 months. All were violent armed offenders. Eighteen of the individuals had committed violent robberies. Thirteen individuals were convicted murderers. Eleven individuals had a criminal record prior to the age of 15. None of them had a sexual offense record.

**Substance Use/Abuse and Medical Records**

Although we sought to recruit a group of relatively “pure” psychopaths, we avoided excessive subject exclusion from associated medical factors in an attempt to maximally provide a generally representative inmate psychopath population. No subject had consumed alcohol (except for one sporadic consumer) or relevant amounts of psychoactive substances for at least two months prior to the assessment (verified using drug-urine testing).

Drug-urine testing was negative for all drugs with the exception of 4 sporadic consumers in which cannabinoids were detected. A total of 5 individuals showed a history of alcohol abuse and 12 individuals had been sporadic consumers of alcohol. Sixteen individuals had a history of other psychoactive substance use for at least one of the following substances: cannabis, opioids, cocaine and other stimulants, hallucinogens and inhalants. Presence of alcohol and/or drug abuse or dependence was registered to be later used as a confounding factor in both behavioral and imaging analyses (0=absence / 1=presence of prior abuse/dependence).

None of the subjects had suffered from any relevant symptomatic medical illness prior to the study. A total of 4 individuals showed positive testing for asymptomatic HIV infection, and 3 additional individuals for asymptomatic hepatitis B and C infections. However, none of them presented neurological complications. Intake of psychotropic medication was also assessed (0=not taking, 1=taking).
