# Supplementary material for: Brain Structural Correlates of Emotion Recognition in Psychopaths
Source: PLoS One. 2016 May 13;11(5):e0149807. doi: 10.1371/journal.pone.0149807 (PMC4866737; doi:10.1371/journal.pone.0149807)
Supplement: S1 Fig — (DOCX) [file pone.0149807.s002.docx]

**Figure S1. Display of the gray matter brain volumes associated with emotional recognition**


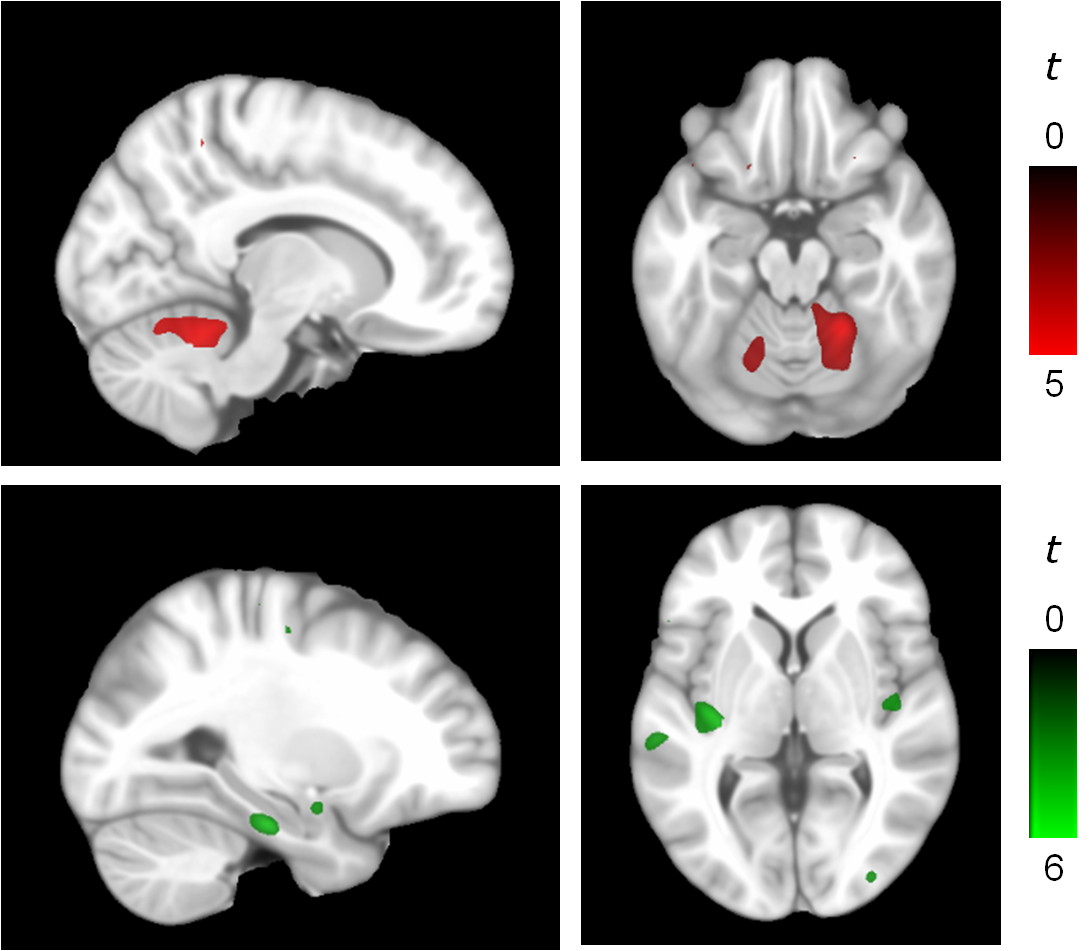


Brain areas in which regional GM volumes were more strongly associated with improved overall emotional recognition in psychopaths vs. control subjects (red) or in control subjects vs. psychopaths (green). The right hemisphere corresponds to the right side in axial displays. The sagittal view corresponds to the right hemisphere for the cerebellum display and to the left hemisphere for the amygdale and parahippocampus display.
