# Supplementary material for: Brain Structural Correlates of Emotion Recognition in Psychopaths
Source: PLoS One. 2016 May 13;11(5):e0149807. doi: 10.1371/journal.pone.0149807 (PMC4866737; doi:10.1371/journal.pone.0149807)
Supplement: S2 Fig — (DOCX) [file pone.0149807.s003.docx]

**Figure S2**. **Associations of PCL-R Facets and gray mater volume, and emotional recognition performance**

**
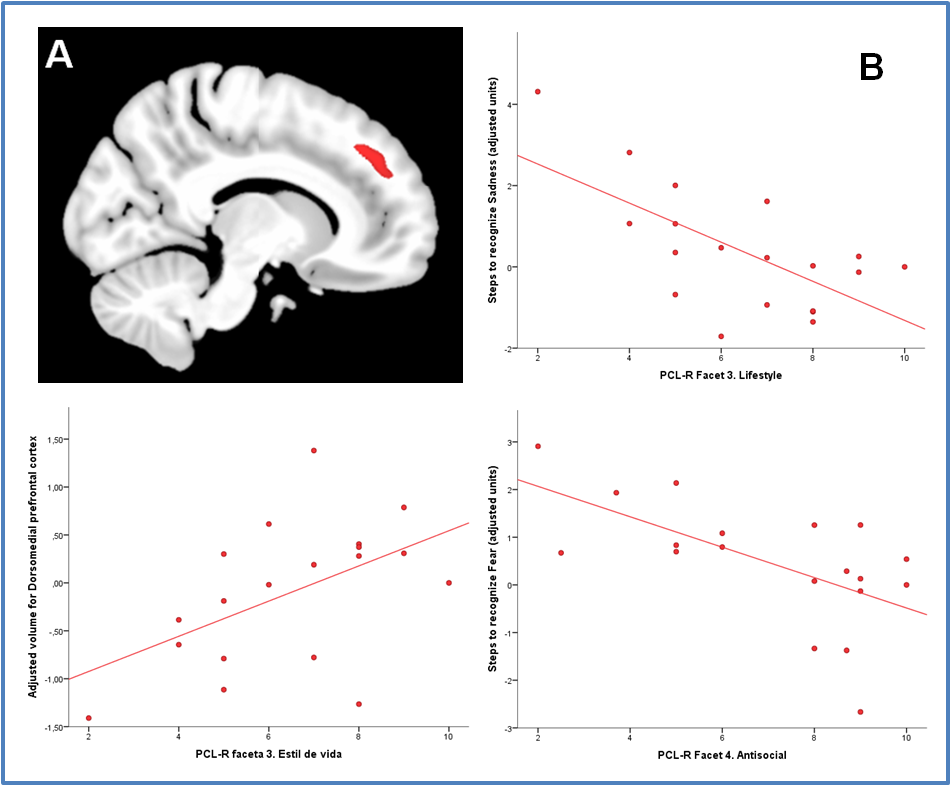
**

Significant associations between PCL-R Facets and (A) dorsomedial prefrontal cortex volume and (B) task performance in psychopaths. The plot displays residual variables once covariates were controlled for.
