# Supplementary material for: Brain Structural Correlates of Emotion Recognition in Psychopaths
Source: PLoS One. 2016 May 13;11(5):e0149807. doi: 10.1371/journal.pone.0149807 (PMC4866737; doi:10.1371/journal.pone.0149807)
Supplement: S1 Table — (DOCX) [file pone.0149807.s004.docx]

**Table S1**. **Pair-wise differences between each emotion category**

| **Emotional expressions** | *Sadness* | *Fear* | *Happiness* | *Surprise* | *Anger* | *Disgust* |
| --- | --- | --- | --- | --- | --- | --- |
|  |  |  |  |  |  |  |
| **Sadness** | - | -0.50+1.89 | 1.83+1.35* | -0.013+2.25 | 0.99+1.75* | -4.72+3.02* |
| **Fear** | 0.50+1.89 | - | 2.33+1.23* | 0.49+1.82 | 1.49+1.52* | -4.22+2.67* |
| **Happiness** | -1.83+1.35* | -2.33+1.23* | - | -1.85+1.61* | -0.85+1.39* | -6.55+2.61* |
| **Surprise** | 0.01+2.25 | -0.49+1.82 | 1.85+1.61* | - | 1.00+ 1.59* | -4.71+2.77* |
| **Anger** | -0.99+1.75* | -1.49+1.52* | 0.85+1.39* | -1.00+1.59* | - | -5.71+2.65* |
| **Disgust** | 4.72+3.02* | 4.22+2.67* | 6.55+2.61* | 4.71+2.77* | 5.71+2.65* | - |
|  |  |  |  |  |  |  |

Paired t-test of the mean number of steps needed to recognize each of the face emotional expressions. Differences of Mean + SD are displayed.

Note: *P<0.05. The mean steps needed to recognize each of the face emotional expressions in columns was compared with the corresponding mean of each of the emotional face expressions in rows (i.e. disgust is much more difficult to recognize than sadness. The positive value in this case indicates that, overall, all subjects in the study require more steps to recognize disgust relative to sadness).
