# Supplementary material for: Brain Structural Correlates of Emotion Recognition in Psychopaths
Source: PLoS One. 2016 May 13;11(5):e0149807. doi: 10.1371/journal.pone.0149807 (PMC4866737; doi:10.1371/journal.pone.0149807)
Supplement: S2 Table — (DOCX) [file pone.0149807.s005.docx]

**S2 Table.** **Brain coordinates of the gray matter brain volumes associated with emotional recognition**

Brain areas in which gray matter volume was more strongly associated with better emotional face expression recognition in psychopaths than in control subjects and brain areas in which gray matter volume was more strongly associated with better emotion recognition performance in control subjects than in psychopaths.

| **Brain Region** | **x, y, z** | **t** | **CS** |
| --- | --- | --- | --- |
| **Controls > Psychopaths** | |  |  |
| Cerebellum | 21 -50 -18 | 5.0 | 2044 |
| **Psychopaths > Controls** | |  |  |
| Amygdala | -20 -3 -17 | 4.4 | 969* |
| Posterior Insula | 45 -9 -2 | 4.1 | 446 |
|  | -35 -20 6 | 5.9 | 1008 |
| Temporal | -59 -29 3 | 4.0 | 325 |
| Parahippocampus | -27 -23 -23 | 4.5 | 969* |

Coordinates (x, y, z) are given in Montreal Neurological Institute (MNI) Atlas space. CS, Cluster size. *same cluster
